# Promoter methylation and expression changes of *BRCA1* in cancerous tissues of patients with sporadic breast cancer

**DOI:** 10.3892/ol.2015.2908

**Published:** 2015-01-27

**Authors:** QIUYUN LI, WEI WEI, YI JIANG, HUAWEI YANG, JIANLUN LIU

**Affiliations:** 1 Department of Breast Surgery, The Affiliated Tumor Hospital of Guangxi Medical University, Guangxi 530021, P.R. China; 2 Department of General Surgery, No. 303 Hospital of PLA, Nanning, Guangxi 530021, P.R. China

**Keywords:** breast cancer, *BRCA1*, DNA methylation

## Abstract

*BRCA1* is a susceptibility gene that has a genetic predisposition for breast cancer. *BRCA1* gene mutation is closely associated with familial hereditary breast cancer, but the *BRCA1* gene mutation is rarely found in sporadic breast cancer. According to previous studies, decreased expression of *BRCA1* was detected in certain types of sporadic breast cancer. Aberrant methylation of DNA promoter CpG islands is one of the mechanisms by which tumor suppressor gene expression and function is lost. The aim of the present study was to investigate *BRCA1* gene expression, methylation status and clinical significance in sporadic types of breast cancer. Quantitative polymerase chain reaction (PCR) and bisulfite sequencing PCR were respectively used to detect expression differences of *BRCA1* mRNA and *BRCA1* methylation in the 49 cancerous and paired non-cancerous samples from patients with breast cancer. The associations of *BRCA1* expression and methylation status with the clinicopathologic characteristics were analysed. *BRCA1* mRNA expression levels in the 49 breast cancer tissues were lower than those in the paired non-cancerous tissues. There was a significant statistical difference (P=0.001). *BRCA1* mRNA expression was not associated with the main clinicopathologic characteristics. Frequency of the *BRCA1* promoter methylation in the breast cancerous tissues was significantly higher than that in the non-cancerous tissues (P=0.007); *BRCA1* gene methylation status was negatively correlated with mRNA expression (P=0.029); and *BRCA1* methylation exhibited no association with all clinicopathological features. DNA promoter hypermethylation may be the potential mechanism accounting for *BRCA1* expression silence in part of sporadic types of breast cancer. Some patients with hypermethylated *BRCA1* may display favorable clinicopathological status.

## Introduction

*BRCA1*, breast cancer susceptibility gene 1, maps to 17q21 (1) and encodes a multifunctional protein involved in DNA repair (2), control of cell-cycle checkpoints (3), ubiquitinylation and chromatin remodeling (4). *BRCA1* was originally identified and cloned as a predisposition gene of familial breast cancer in 1994 (5). Although a significant fraction of familial types of breast cancer could be explained by the inherited mutations of *BRCA1*, a large proportion of familial and sporadic types of breast cancer are not associated with mutations in *BRCA1* (6–9) Furthermore, *BRCA1* mRNA levels were also found to be reduced or absent in invasive sporadic types of breast cancer, thus assigning a role of *BRCA1* in these as well (10–12). This suggests that other mechanisms for loss of functions may exist.

Breast cancer results from the manifestation of genetic and epigenetic changes in tumor suppressor genes and oncogenes (13,14). Although the causal association remains under debate, increasing evidence has shown that hypermethylation of promoter CpG islands (15,16), accompanied by global hypomethylation (17,18), are common molecular events in cancer cells. Promoter CpG islands, which frequently locate at the 5′ end regulatory regions of genes, are subject to epigenetic modification by DNA methylation which is known to play an important role in regulating gene expression (16,19). If promoter CpG islands of key genes were hypermethylated and form a closed repressive chromatin configuration, the transcription initiation of the corresponding genes should be affected (20).

There are reports that *BRCA1* promoter methylation status is associated with downregulated mRNA and protein levels in breast cancerous tissues (21,22) and cell lines (23). Aberrant *BRCA1* promoter methylation is associated with particular biological and clinicopathological features (24,25). However, these studies failed to lead to a conclusive finding. In the current study, the hypothesis is that the absence of *BRCA1* transcript is associated with promoter methylation in sporadic types of breast cancer. The present study further investigates *BRCA1* gene expression, methylation status and their clinical significance in sporadic breast cancer.

## Materials and methods

### Study cohort and tissue samples

The study was approved by the ethics committee of Guangxi Medical University (Nanning, China). All patients involved in the study provided their informed consent. The study cohort consisted of 49 patients, who were randomly selected from patients continuously diagnosed with operable breast cancer between September 2010 and September 2012 in the Department of Breast Surgery of the Affiliated Tumor Hospital of Guangxi Medical University. Patients were excluded from participation in the case of familial types of breast cancer; prior chemotherapy or radiotherapy for any malignancy; and pregnancy or lactation.

All the studied samples included 49 surgically resected cancerous tissues and 49 corresponding paired non-cancerous tissues which were taken >5 cm from the tumor macroscopically (in cases where such distance was not present, the non-cancerous sample was taken from the distance furthest from the tumor sample). These samples were the fresh tissues following surgical removal, and were immediately put into liquid nitrogen for 10 min and then into a −80°C ultra freezer. All samples were subsequently reviewed and confirmed by the Department of Pathology of the Affiliated Tumor Hospital of Guangxi Medical University. Pathological information was collected from the patient clinical database, and the information was blinded in another database. The clinicopathologic characteristics of patients included histological tumor type, primary tumor size, axillary nodal status, grade of the disease, estrogen/progesterone receptor (ER/PR) status or HER-2/neu status.

### RNA extraction and quantitative polymerase chain reaction (PCR)

The RNA isolated from the breast cancerous tissues and paired non-cancerous tissues were kept using TRIzol^®^ reagent (Invitrogen Life Technologies, Carlsbad, CA, USA) according to the manufacturer’s instructions. β-actin mRNA was the reference gene used as the internal control. The primers of *BRCA1* and β-actin (Invitrogen Life Technologies) are shown in Table I. The PCR cycle conditions used are 95°C for 2 min; 40 cycles at 95°C for 10 sec, 60°C for 30 sec, and 70°C for 30 sec; and final extension at 72°C for 7 min. Dissociation curve analyses were used to confirm the specificity of the SYBR^®^ Green (Invitrogen Life Technologies) signals in each experiment. Data were analyzed using ABI Prism 7900 SDS software (Applied Biosystems, Waltham, MA, USA). The mRNA expression of *BRCA1* was analyzed using the 2^−ΔΔCt^ method (26). Fluorescent data were converted into RQ measurements, which stand for relative expression automated by the system software. Thermal dissociation plots were examined for biphasic melting curves. To ensure experiment accuracy, quantitative PCR products were randomly selected for sequencing.

### DNA extraction and sodium bisulfite modification

Total genomic DNA of the specimens were isolated from the breast cancerous tissues and paired non-cancerous tissues, by the DNeasy Tissue AxyPrep DNA extraction kit (Tiangen, Beijing, China). All procedures were followed according to the manufacturer’s instructions. Genomic DNA was modified with bisulfite using MethylCode™ Bisulfite Conversion kit (Invitrogen Life Technologies) according to the manufacturer’s instructions.

### Bisulfite genomic sequencing

Bisulfite genomic DNA sequencing was carried out as previously described (27) with sodium bisulfite modification. The CpG islands of promoter region located between −937 and −717 bp (translation start site as 1). The bisulfite-treated DNA was subjected to PCR in order to amplify the *BRCA1* promoter region. The primers of bisulfite genomic sequencing are shown in Table I. PCR products were purified and cloned into the pMD18-T vector (Takara, Dalian, China), then transformed into *Escherichia coli* strain DH5α (Invitrogen Life Technologies). Five positive clones for each sample were selected and analyzed using the ABI 3730 DNA Sequencer (Applied Biosystems). The percentage of methylation for each sample was calculated as the number of methylated CpG dinucleotides/(5×48) × 100%.

### Statistical analysis

Statistical analysis was performed using SPSS software version 13.0 (SPSS, Inc., Chicago, IL, USA). Gene expression levels or DNA methylation status of paired samples with normal distribution were expressed as the mean ± standard deviation; otherwise, they were expressed as the median with the first and third interquartile ranges (IQR1 and IQR3). Associations between *BRCA1* mRNA expression or DNA methylation and the categorical variables were assessed by the Pearson’s χ^2^ or Mann-Whitney U tests, as appropriate. Correlation coefficients were assessed by Spearman’s correlation analysis. P<0.05 was considered to indicate a statistically significant difference, and all P-values were two-sided.

## Results

### Expression of BRCA1 in breast cancerous and paired non-cancerous samples

**Figure 1:**
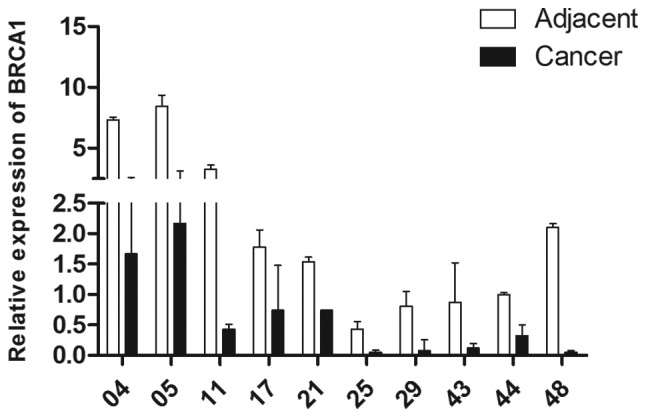
Relative expression of *BRCA1* in breast cancer and paired adjacent non-cancerous samples from the selected patients. *BRCA1* expression was expressed as the 2^−ΔΔCT^.

In the present study, the median level of *BRCA1* in non-cancerous samples was set as 1. The median RQs of *BRCA1* mRNA in breast cancerous and paired non-cancerous samples were 0.33 (IQR1, 0.18; IQR3, 0.95) and 0.94 (IQR1, 0.46; IQR3, 1.98), respectively. The difference between the two group was statistically significant (Wilcoxon matched-pairs signed-ranks test, P=0.001). The representative results of the quantitative PCR are provided in Fig. 1. The results indicate that the expression of *BRCA1* in breast cancer was aberrantly decreased at the transcriptional level.

According to the median RQ of the paired non-cancerous tissues which was 0.94, the tumor tissues were divided into three groups: overexpression (>0.94), normal expression (=0.94) and reduced expression (<0.94). Due to the limited number of tissues in the over and normal expression groups, these two groups were combined into one group, named the unreduced expression group. The correlation between *BRCA1* mRNA and the main clinicopathologic characteristics was also analyzed. The associations between them are shown in Table II. No significant correlation was observed between *BRCA1* mRNA and the various parameters.

### Correlation of BRCA1 expression and methylation in breast cancerous and paired non-cancerous samples

**Figure 2:**
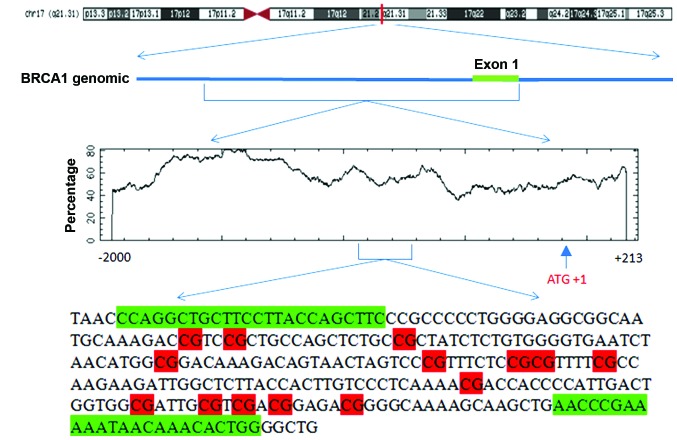
Genomic architecture of the human *BRCA1* gene. (A) Location of the *BRCA1* gene within human chromosome 17 (ch17 q21.31); (B) exon/intron structure of the human *BRCA1* gene. Noted is the relative location of the first 1 coding exons and the translational start (ATG) codons. (C) Structure of 5′ end of *BRCA1* gene. Graph of percent guanine (G) and cytosine (C) nucleotides across this region and boundaries of the CpG island. (D) Detailed information of the *BRCA1* promoter region sequence. The bisulfite sequencing polymerase chain reaction primers are presented in the green shaded region. There are 14 CpG sites in this region.

Analysis was carried out using the Methyl Primer Express version 1.0 (Applied Biosystems) to analyze the CpG islands of the region between −2,000 and +1,000 bp, including the translational initiation codon (ATG) in detail. In the 5′ end of the *BRCA1* gene, two CpG islands were revealed, a 244 bp (between −1,279 and −1,036 bp) and a 221 bp (between −937 and −717 bp) segment (Fig. 2).

**Figure 3:**
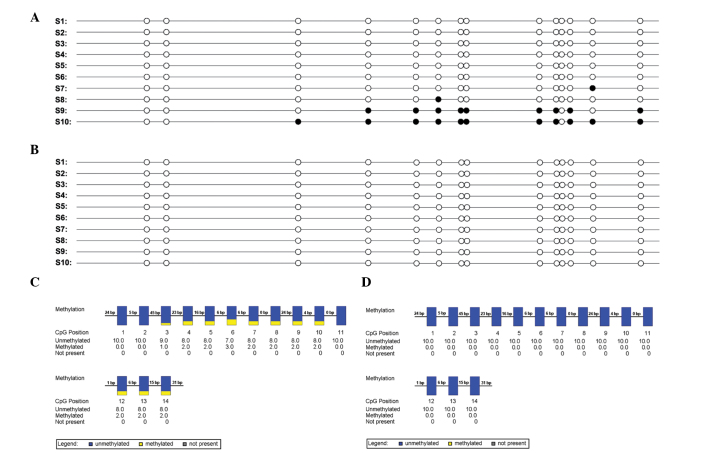
Methylation status analysis of *BRCA1* in breast cancer. BSP methylation status analysis of *BRCA1* in (A) breast cancer and (B) non-cancerous tissues; Sulfite process BSP sequencing mode of *BRCA1* in (C) breast cancer and (D) non-cancerous tissues. BSP, bisulfite sequencing polymerase chain reaction.

To determine whether epigenetic silencing of the *BRCA1* gene also occurs in primary breast cancer, the *BRCA1* methylation status in 49 paired breast cancer and corresponding non-cancerous tissues was examined (Fig. 3). Aberrant hypermethylation was detected in 24 of 49 (49%) tumors, which was more frequent than that in the paired no-cancerous tissues (11 of 49, 22.4%; Wilcoxon matched-pairs signed-ranks test, P=0.007). In 24 cases of hypermethylation of cancerous tissues, 20 (83.3%) showed a lower *BRCA1* mRNA expression. Furthermore, it was revealed that the association between *BRCA1* mRNA expression level and methylation status was a negative correlation (r=-0.311, P=0.029), which indicated a correlation between CpG island hypermethylation and transcriptional silencing.

### Association between BRCA1 methylation level in breast cancer and the main clinicopathological parameters

To ascertain the potential clinical significance of the epigenetic event, analysis was conducted on the main clinicopathological characters and methylation status of *BRCA1* in the 49 cases. The associations between *BRCA1* methylation status and various clinicopathological parameters are shown in Table III. No significant correlation was observed between *BRCA1* hypermethylation and main parameters such as age at diagnosis, menopausal status, tumor, node and metastasis (TNM) stage, primary tumor size, axillary nodal status, ER/PR status or HER-2/neu status.

## Discussion

*BRCA1* is a well-established breast cancer susceptibility gene, and is involved in maintaining genome integrity through pathways including participation in DNA damage repair, the control of cell cycle checkpoints and apoptosis (2–4). In these functions, *BRCA1* is implicated in the repair of double strand DNA breaks by homologous chromosomal recombination (28,29). Deficiencies in homology-directed DNA repair cause high levels of genomic instability that increases the risk of tumorigenesis (30). *BRCA1* that impairs such function leads to increased proliferation and chromosomal instability. It has been proved that *BRCA1* mutation is one of the main genetic events in the hereditary type of breast cancer (6), but no or limited somatic mutations in *BRCA1* have been found in the sporadic form of breast cancer. On the another hand, a growing number of studies have demonstrated loss of heterozygosity and a reduced level or absence of *BRCA1* expression in sporadic breast cancer (31,32). These two factors suggest that transcriptional and/or posttranscriptional repression of *BRCA1* may participate in the development of sporadic breast cancer. One of the common mechanisms of functional inactivation of tumor suppressor genes in cancer cells is the aberrant DNA hypermethylation of CpG islands in the promoter region of the gene that is associated with the loss of gene expression.

Firstly, *BRCA1* expression at the mRNA level was detected in paired cancerous and non-cancerous tissue of sporadic breast cancer. *BRCA1* expression of breast cancerous tissues showed a relatively lower level as compared with those of the paired non-cancerous tissues. The difference between them was statistically significant. The data indicated that the expression of *BRCA1* in breast cancer was aberrantly reduced. Subsequently, the present study demonstrated that the low expression of *BRCA1* was significantly correlated with the hypermethylation in its promoter region. In the present study, *BRCA1* hypermethylation was detected in 49% of the cases, which was consistent with other previous reports (9.1~59%) (33–35). The differences in the frequency of hypermethylation among the studies may be accounted for by several factors including: Methodology, study cohort, adjacent non-cancerous tissues contaminated by cancer cells and population differences due to exposure to specific environmental factors.

Furthermore, the correlation between *BRCA1* hypermethylation and the main clinicopathological characters was analyzed. Ever since *BRCA1* hypermethylation was proved to be involved in sporadic breast cancer, some studies were dedicated to explore the correlation between its aberrant methylation and the disease characteristics. *BRCA1* promoter methylation status displayed various disease characteristic phenotypes in different studies; however, the majority of studies demonstrated that *BRCA1* hypermethylation correlated with lack of estrogen and progesterone receptor expression in younger females (<50 years). Nevertheless, the present study did not discover a significant association between *BRCA1* hypermethylation and ER/PR status. This result was similar to that reported in a previous study by Xu *et al* (36). Furthermore, in the study by Matros *et al* (37), they even found that *BRCA1* hypermethylation is correlated with progesterone receptor positive expression, suggesting a more complex phenotypic association.

In addition, two interesting details were revealed which may be associated with favorable clinical prognosis, though there was no association between *BRCA1* hypermethylation and the main clinicopathological characters including age at diagnosis, menopausal status, TNM stage, primary tumor size, axillary nodal status, ER/PR status or HER-2/neu status in sporadic breast cancer. Firstly, the *BRCA1* hypermethylation exhibited a higher percentage of the smaller size primary tumor (T1 and T2, tumor size ≤5 cm) compared to the *BRCA1* non-methylation ( 58.1% vs. 49.1%). The result seemed to display a trend that *BRCA1* hypermethylation tumors tended to be the smaller tumor size. The larger size of tumor is one of the most important indicators for poor prognosis. Secondly, there was more *BRCA1* hypermethylation of low Ki-67 index (<15%) cancerous tissues compared with the *BRCA1* non-methylation (60% vs. 40%). The high Ki-67 index (≥15%), which is one of the important parameters for luminal phenotype, has been proven to correlate with a greater carcinogenic aggressiveness and worse prognosis. The reasons underlying the phenomenon of *BRCA1* methylation were not elucidated, but some evidence was found correlating *BRCA1* hypermethylation and favorable disease characteristics in a study by Li *et al* (38). On the basis of a smaller sample the study demonstrated high survival rates associated with *BRCA1* hypermethylation. Krasteva *et al* (39) also reported that breast cancer with *BRCA1* hypermethylation was associated with improved overall survival rates. Those evidences may partly explain the present findings. Following cautious consideration, the findings from the present study do not appear to be contradictory to previous studies. By contrast, the present study results once again manifested that breast cancer was a type of heterogeneous disease from one aspect.

In conclusion, the present study revealed that *BRCA1* expression was expressed at low levels in the majority of sporadic breast cancerous tissues, and DNA promoter hypermethylation may be the potential mechanism accounting for *BRCA1* expression silence. Secondly, the reduced *BRCA1* expression and *BRCA1* hypermethylation did not correlate with any clinicopathological features. Finally, partial sporadic breast cancer with *BRCA1* hypermethylation may exhibit favorable clinicopathological status. It is thus reasonable to explore *BRCA1* epigenetic inactive mechanism and identify a subset of sporadic breast cancer with a specific epigenetic phenotype. Further studies to observe whether a specific *BRCA1*-related sporadic breast cancer can indicate a favorable prognosis would be beneficial.

## Figures and Tables

**Table I:** Primer sequences used in the study.

Gene/primer	Sequence
*BRCA1*	
Forward	TGTGAGGCACCTGTGGTGAC
Reverse	GTGGCTGGCTGCAGTCAGTAG
β-catenin	
Forward	GAAACGGCTTTCAGTTGAGC
Reverse	CTGGCCATATCCACCAGAGT
Bisulfite sequencing primer	
Forward	GATTGGGTGGTTAATTTAGAGT
Reverse	AATTATCTAAAAAACCCCACAA

**Table II:** Correlations between *BRCA1* mRNA expression and the main clinicopathologic characteristics.

		*BRCA1* mRNA expression				
			
Variable	n	Reduced	%	Overexpression	%	P-value
Age (years)						0.304^a^
<50	28	19	67.9	9	32.1	
≥50	21	17	81.0	4	19.0	
Menopause						0.129^a^
Pre	29	19	65.5	10	34.5	
Post	20	17	85.0	3	15.0	
TNM stage						0.078^b^
I	9	5	55.6	4	44.4	
II	24	17	70.8	7	29.2	
III	16	14	87.5	2	12.5	
ER						0.219^a^
Negative	14	12	85.7	2	14.3	
Positive	35	24	68.6	11	31.4	
PR						0.232^a^
Negative	22	18	81.9	4	18.2	
Positive	27	18	66.7	9	33.3	
HER-2/neu						0.156^a^
Negative	34	27	79.4	7	20.6	
Positive	15	9	60.0	6	40.0	
Ki-67						0.492^a^
<0.15	15	12	80.0	3	20.0	
≥0.15	34	24	70.6	10	29.4	
Axillary nodes						0.682^a^
Negative	24	17	70.8	7	29.2	
Positive	25	19	76.0	6	24.0	
Tumor stage						0.140^b^
T1	6	3	50.0	3	50.0	
T2	25	18	72.0	7	28.0	
T3	12	10	83.3	2	16.7	
T4	6	5	83.3	1	16.7	

a P-value when expression levels were compared using the Pearson’s χ^2^ test.

b P-value when expression levels were compared using the Mann-Whitney U test.

TNM, tumor, node and metastasis; ER, estrogen receptor; PR, progesterone receptor.

**Table III:** Correlations between *BRCA1* methylation status and the main clinicopathological characteristics.

		*BRCA1* methylation status				
			
Variable	n	Reduced	%	Overexpression	%	P-value
Age						0.458^a^
<50	28	13	46.4	15	53.6	
≥50	21	12	57.1	9	42.9	
Menopause						0.644^a^
Pre	29	14	48.3	15	51.7	
Post	20	11	55.0	9	45.0	
TNM stage						0.465^b^
I stage	9	4	44.4	5	55.6	
II stage	24	15	62.5	9	37.5	
III stage	16	6	37.5	10	62.5	
ER						0.470^a^
Negative	14	6	42.9	8	57.1	
Positive	35	19	54.3	16	45.7	
PR						0.201^a^
Negative	22	9	40.9	13	59.1	
Positive	27	16	59.3	11	40.7	
HER-2/neu						0.686^a^
Negative	34	18	52.9	16	47.1	
Positive	15	7	46.7	8	53.3	
Ki-67						0.086^a^
<0.15	15	6	40.0	9	60.0	
≥0.15	34	19	55.9	15	44.1	
Axillary nodes						0.666^a^
Negative	24	13	54.2	11	45.9	
Positive	25	12	48.0	13	52.0	
Tumor stage						0.508^b^
T1	6	2	33.3	4	66.7	
T2	25	11	44.0	14	56.0	
T3	12	9	75.0	3	25.0	
T4	6	3	50.0	3	50.0	

a P-value when expression levels were compared using the Pearson’s χ^2^ test.

b P-value when expression levels were compared using the Mann-Whitney U test.

TNM, tumor, node and metastasis; ER, estrogen receptor; PR, progesterone receptor.

## References

[1] Hall JM, Lee MK, Newman B (1990). Linkage of early-onset familial breast cancer to chromosome 17q21. Science.

[2] Deng CX, Wang RH (2003). Roles of BRCA1 in DNA damage repair: a link between development and cancer. Hum Mol Genet.

[3] Xu B, St K, Kastan MB (2001). Involvement of BRCA1 in S-phase and G(2)-phase checkpoints after ionizing irradiation. Mol Cell Biol.

[4] Ralhan R, Kaur J, Kreienberg R, Wiesmüller L (2007). Links between DNA double strand break repair and breast cancer: accumulating evidence from both familial and nonfamilial cases. Cancer Lett.

[5] Miki Y, Swensen J, Shattuck-Eidens D (1994). A strong candidate for the breast and ovarian cancer susceptibility gene BRCA1. Science.

[6] Easton DF, Bishop DT, Ford D, Crockford GP (1993). Genetic linkage analysis in familial breast and ovarian cancer: results from 214 families. The Breast Cancer Linkage Consortium. Am J Hum Genet.

[7] Narod SA, Ford D, Devilee P (1995). An evaluation of genetic heterogeneity in 145 breast-ovarian cancer families. Breast Cancer Linkage Consortium. Am J Hum Genet.

[8] Ford D, Easton DF, Stratton M (1998). Genetic heterogeneity and penetrance analysis of the BRCA1 and BRCA2 genes in breast cancer families. The Breast Cancer Linkage Consortium. Am J Hum Genet.

[9] Hedenfalk I, Ringner M, Ben-Dor A (2003). Molecular classification of familial non-BRCA1/BRCA2 breast cancer. Proc Natl Acad Sci USA.

[10] Thompson ME, Jensen RA, Obermiller PS, Page DL, Holt JT (1995). Decreased expression of BRCA1 accelerates growth and is often present during sporadic breast cancer progression. Nat Genet.

[11] Magdinier F, Ribieras S, Lenoir GM, Frappart L, Dante R (1998). Down-regulation of BRCA1 in human sporadic breast cancer; analysis of DNA methylation patterns of the putative promoter region. Oncogene.

[12] Bianco T, Chenevix-Trench G, Walsh DC, Cooper JE, Dobrovic A (2000). Tumour-specific distribution of BRCA1 promoter region methylation supports a pathogenetic role in breast and ovarian cancer. Carcinogenesis.

[13] Jones PA, Baylin SB (2002). The fundamental role of epigenetic events in cancer. Nat Rev Genet.

[14] Franco R, Schoneveld O, Georgakilas AG, Panayiotidis MI (2008). Oxidative stress, DNA methylation and carcinogenesis. Cancer Lett.

[15] Herman JG, Baylin SB (2003). Gene silencing in cancer in association with promoter hypermethylation. N Engl J Med.

[16] Das PM, Singal R (2004). DNA methylation and cancer. J Clin Oncol.

[17] Feinberg AP, Vogelstein B (1983). Hypomethylation distinguishes genes of some human cancers from their normal counterparts. Nature.

[18] Choi JY, James SR, Link PA (2009). Association between global DNA hypomethylation in leukocytes and risk of breast cancer. Carcinogenesis.

[19] Robertson KD (2002). DNA methylation and chromatin-unraveling the tangled web. Oncogene.

[20] Baylin SB, Ohm JE (2006). Epigenetic gene silencing in cancer-a mechanism for early oncogenic pathway addiction. Nat Rev Cancer.

[21] Esteller M, Silva JM, Dominguez G (2000). Promoter hypermethylation and BRCA1 inactivation in sporadic breast and ovarian tumors. J Natl Cancer Inst.

[22] Birgisdottir V, Stefansson OA, Bodvarsdottir SK, Hilmarsdottir H, Jonasson JG, Eyfjord JE (2006). Epigenetic silencing and deletion of the BRCA1 gene in sporadic breast cancer. Breast Cancer Res.

[23] Rice JC, Massey-Brown KS, Futscher BW (1998). Aberrant methylation of the BRCA1 CpG island promoter is associated with decreased BRCA1 mRNA in sporadic breast cancer cells. Oncogene.

[24] Catteau A, Harris WH, Xu CF, Solomon E (1999). Methylation of the BRCA1 promoter region in sporadic breast and ovarian cancer: correlation with disease characteristics. Oncogene.

[25] Turner N, Tutt A, Ashworth A (2004). Hallmarks of ‘BRCAness’ in sporadic cancers. Nat Rev Cancer.

[26] Livak KJ, Schmittgen TD (2001). Analysis of relative gene expression data using real-time quantitative PCR and the 2(−Delta Delta C(T)) Method. Methods.

[27] Chu D, Zhang Z, Li Y (2011). Prediction of colorectal cancer relapse and prognosis by tissue mRNA levels of NDRG2. Mol Cancer Ther.

[28] Zhang J, Powell SN (2005). The role of the BRCA1 tumor suppressor in DNA double-strand break repair. Mol Cancer Res.

[29] Ting NS, Lee WH (2004). The DNA double-strand break response pathway: becoming more BRCAish than ever. DNA Repair (Amst).

[30] De Vargas Roditi L, Michor F (2011). Evolutionary dynamics of BRCA1 alterations in breast tumorigenesis. J Theor Biol.

[31] Valentin MD, da Silva SD, Privat M, Alaoui-Jamali M, Bignon YJ (2012). Molecular insights on basal-like breast cancer. Breast Cancer Res Treat.

[32] Warmoes M, Jaspers JE, Pham TV (2012). Proteomics of mouse BRCA1-deficient mammary tumors identifies DNA repair proteins with potential diagnostic and prognostic value in human breast cancer. Mol Cell Proteomics.

[33] Esteller M, Silva JM, Dominguez G (2000). Promoter hypermethylation and BRCA1 inactivation in sporadic breast and ovarian tumors. J Natl Cancer Inst.

[34] Rice JC, Ozcelik H, Maxeiner P, Andrulis I, Futscher BW (2000). Methylation of the BRCA1 promoter is associated with decreased BRCA1 mRNA levels in clinical breast cancer specimens. Carcinogenesis.

[35] Krasteva ME, Bozhanov SS, Antov GG, Gospodinova ZI, Angelov SG (2012). Breast cancer patients with hypermethylation in the promoter of BRCA1 gene exhibit favorable clinical status. Neoplasma.

[36] Xu X, Gammon MD, Zhang Y (2009). BRCA1 promoter methylation is associated with increased mortality among women with breast cancer. Breast Cancer Res Treat.

[37] Matros E, Wang ZC, Lodeiro G, Miron A, Iglehart JD, Richardson AL (2005). BRCA1 promoter methylation in sporadic breast tumors: relationship to gene expression profiles. Breast Cancer Res Treat.

[38] Li S, Rong M, Iacopetta B (2006). DNA hypermethylation in breast cancer and its association with clinicopathological features. Cancer Lett.

[39] Krasteva ME, Bozhanov SS, Antov GG, Gospodinova ZI, Angelov SG (2012). Breast cancer patients with hypermethylation in the promoter of BRCA1 gene exhibit favorable clinical status. Neoplasma.

